# An initial industrial flora: A framework for botanical research in cooperation with industry for biodiversity conservation

**DOI:** 10.1371/journal.pone.0230729

**Published:** 2020-04-01

**Authors:** Rima D. Lucardi, Chelsea E. Cunard, Steven C. Hughes, Kevin S. Burgess, Jennifer N. Reed, Lauren E. Whitehurst, Samantha J. Worthy, Travis D. Marsico

**Affiliations:** 1 Forest Service, United States Department of Agriculture (USDA), Athens, Georgia, United States of America; 2 Department of Biological Sciences, Arkansas State University, Jonesboro, Arkansas, United States of America; 3 Department of Plant Biology, The Herbarium at the University of Georgia, University of Georgia, Athens, Georgia, United States of America; 4 Department of Biology, Columbus State University, Columbus, Georgia, United States of America; Universidad Mayor de San Andrés, PLURINATIONAL STATE OF BOLIVIA

## Abstract

Humans have created an accelerating, increasingly connected, globalized economy, resulting in a more globalized, shared flora. The prevention of new, establishing species is less costly, both economically and ecologically, and is more manageable than eradicating nonnative invasive species once they are widespread and negatively impactful. We ask if international trade hubs and points-of-entry with high-volume trade, constant disturbance, and propagule rain have a higher number of nonnative species compared to surrounding areas and if they may serve as initial establishment sites and refugia of nonnative, invasive populations. Therefore, we partnered with various federal, state, and private interests to evaluate the floristic composition at the Garden City Terminal of the Port of Savannah, Georgia, USA. We conducted the following study to demonstrate the collaborative relationship-building between researchers and industry and to develop a framework for biodiversity conservation. In our study, we collected all reproductive vascular plants in the secured areas of the Garden City Terminal during four major seasonal time points over two years. The percent of nonnative species and number of nonnative plant species per hectare at this industrial location exceeded all other comparison floras. The mean coefficient of conservatism was lowest among the comparison floras, indicating a highly disturbed habitat with nonnative, weedy native, and other native species tolerant of disturbance. Our study represents one of the first inventories of an *Industrialized Flora* and indicates that such areas are hot-spots of nonnative plant diversity and possible sources of emergent plant invasions. We posit that industrial sites and international points-of-entry should be considered laboratories for research on species transport and introduction, adaptability, and taxonomic delineation to better understand the mechanisms and consequences of biotic homogenization due to the volume and frequency of anthropogenic activities.

## Introduction

Research on changes in plant species distributions would not be possible without access to historical and contemporary deposits of quality specimens and data housed in herbaria [1]. These collections are critical resources that serve as a repository for evaluating species’ native range, habitat requirements or preferences, changes in phenology, and for developing conservation plans and prioritizing limited resources [1, 2, 3, 4, 5]. Traditionally, floristic inventories have been used to understand the ecological and evolutionary significance of plant species distributions as well as the conservation implications associated with plant species composition and diversity. Although such inventories have primarily focused on natural areas (see for example, S1 Table), there is increasing evidence that the urbanization of local floras can facilitate increased nonnative plant richness and concomitant extirpation of native species [6]. Commercial transportation routes and their relationship with nonnative plant establishment is a research field that has been expanding in the United States of America (USA; Table 1; [7, 8, 9]) and Europe [10, 11, 12, 13]. These studies have focused on inventorying nonnative plants along transportation corridors and understanding international trade, propagule pressure, and its contribution to invasions. From this body of literature, it is clear that increasing global connectivity associated with human consumption of resources contributes to increased invasive species risk and biotic homogenization [14, 15, 16]. We identified a need for plant species monitoring to assess threats posed by nonnative species on local, regional, and global biodiversity as a result of high-volume anthropogenic exchange of goods, such as pathways well-worn by shipping [17]. We initiated cooperative research inclusive of universities, federal and state agencies, and public and private interests to conduct a floristic inventory of the Garden City Terminal (GCT) at the Port of Savannah, Georgia, USA.

**Table 1:** Reference table of acronyms used throughout this manuscript.

Acronym	Definition	Category/Descriptor
**ANOVA**	Analysis of Variance	Statistic
**APHIS**	Animal-Plant Health Inspection Services	Federal Government Agency within USDA
**COLS**	The Herbarium at Columbus State University	Official Herbarium Designation
**GA**	Georgia	State in the USA
**GCT**	Garden City Terminal	Shipping container handling facility at the Port of Savannah
**PPQ**	Plant Pest Quarantine	Regulatory Division of Federal Government Agency
**SC**	South Carolina	State in the USA
**STAR**	Arkansas State University Herbarium	Official Herbarium Designation
**TEU**	Trade Equivalent Unit	Measure of trade volume
**USA**	United States of America	Geographic/Political Area
**USCBP**	United States Customs & Border Protection	Federal Government Agency, Department of Homeland Security
**USDA**	United States Department of Agriculture	Federal Department

### Environmental science and industry: Natural bedfellows?

**Fig 1:**
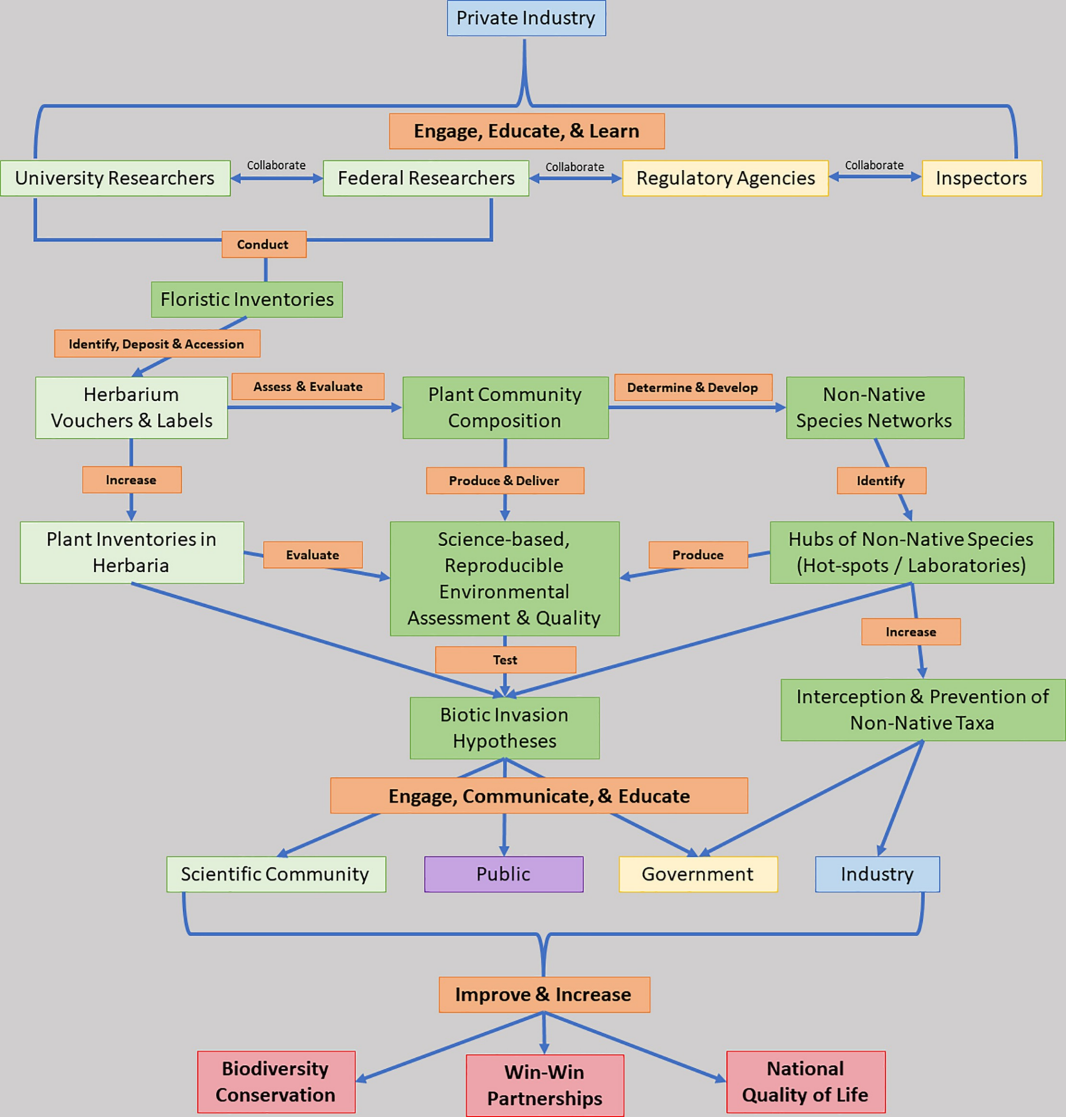
Framework for cooperative interaction across sectors for the purpose of generating meaningful outcomes from *Industrialized Flora* research at ports-of-entry and industrial sites.

Promoting positive partnerships between private industry and research is vital to evaluate multi-faceted environmental issues and to address limited global resources in the face of increasing public needs (Fig 1). Here, we advocate for the development and emergence of *Industrialized Floras* as a valuable line of research toward the shared goals of global biodiversity conservation and concurrent growth of natural history collections and associated data. Specifically, *Industrialized Floras* are conducted on commercial manufacturing complexes and other private sites associated heavily with international import and export of raw to finished commodities, including agricultural commercial sites. We aim to bridge the scientific knowledge gap of species distributions associated with anthropogenic localities and to deliver high-quality, evidence-based, reproducible science to private industry and the public. Private industry, naturally, has a vested fiscal interest in research and development, as well as creating a balance between market needs, profitability, and access to the raw materials necessary for products and services. Therefore, by linking the goals of environmental science with that of the public and private sector, we aim to demonstrate that our case study represents a nexus of partnerships necessary for the prevention and potential mitigation of the spread of nonnative, invasive plant species from industrial sites (Fig 1).

For the study of *Industrialized Floras*, we are most interested in creating biodiversity inventories of the greenspaces located within industrial complexes that are primarily paved and experience high anthropogenic activity and interchange. Through the creation of a plant inventory of the *Industrialized Flora* at an international seaport, we demonstrate that nonnative plant species are more frequently encountered than at other inventoried sites. Therefore, industrial sites represent potential source populations of nonnative propagules that may spread from sites of introduction into areas of conservation interest or the broader landscape habitat matrix. Industries, regulatory agencies, and environmental scientists all wish to deliver high-quality products and services to their consumers; therefore, it is important for science and industry to work together to inventory plant biodiversity, monitor changes over time, and seek to protect our shared resources (Fig 1).

### International points-of-entry: Gateways for nonnative species introduction and establishment

**Fig 2:**
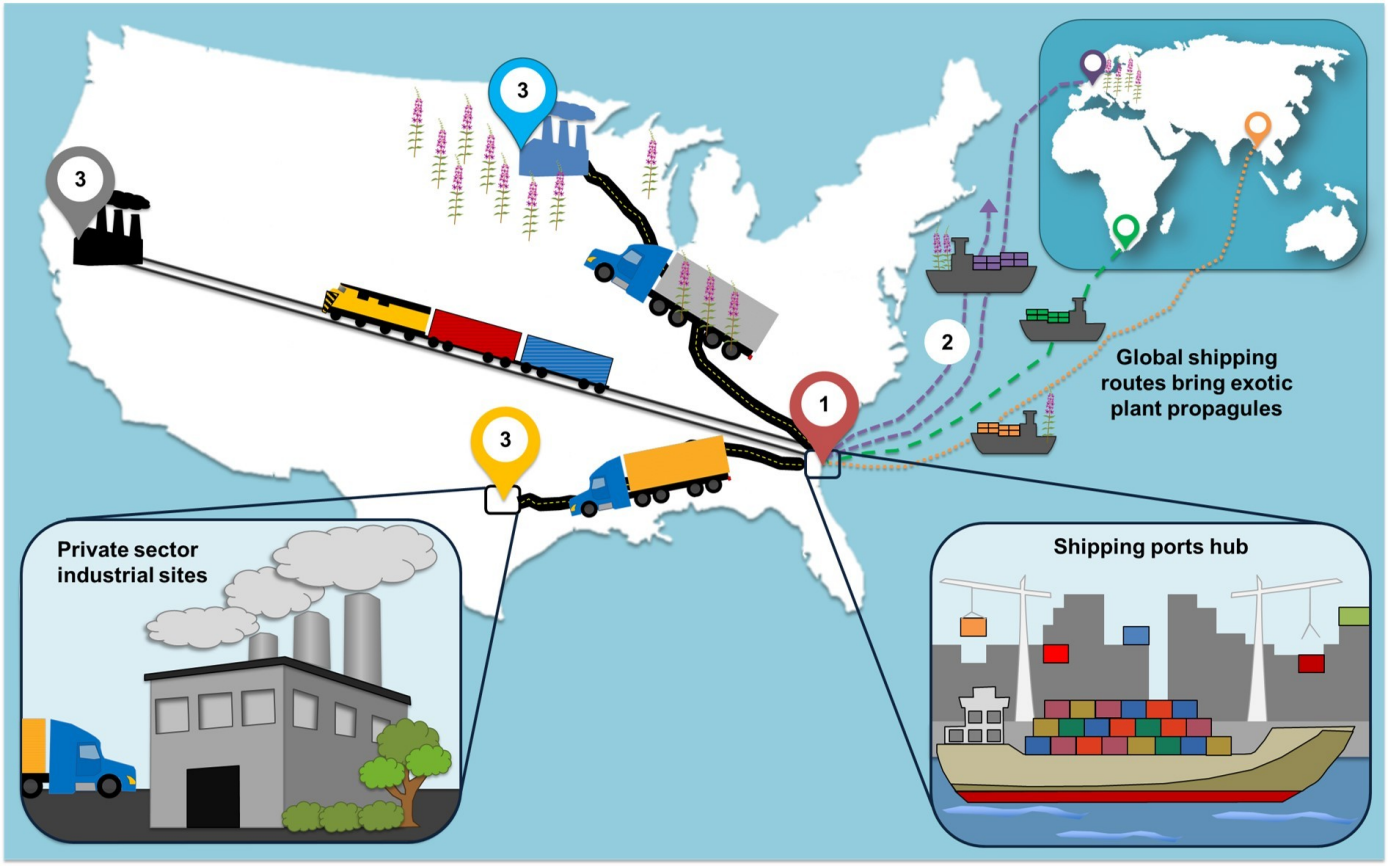
With a concerted effort, botanists should begin monitoring and researching at industrial sites to determine propagule pressure, environmental conditions, disturbance regimes, connectivity among sites, botanical species richness, and invasive species abundance to assess environmental risk of species invasion that current practices allow or promote. Through international trade, (1) global shipping routes bring exotic plant propagules to ports that function as hubs of industrial activity. (2) Sometimes through effective monitoring and detection, contaminated shipments are turned away and sent back to the nation of shipment origin. (3) Upon arrival, shipments (including those that are possibly contaminated) are rapidly sent up to thousands of kilometers away from the port-of-origin to private-sector industrial and commercial sites. Figure created by Ashley N. Schulz.

Points-of-entry, such as seaports and airports, experience the initial interception of international goods and may be the most vulnerable to introductions of nonnative species [18, 19]. From points-of-entry to secondary and tertiary locations, a network of connectivity for plant invasions can develop (Fig 2). Sites with more connectivity to ports, in terms of frequency and volume, intrinsically possess a higher risk of nonnative propagule establishment [17, 20] than those more isolated or further down the supply chain. Points-of-entry typically experience constant human activity coupled with intense terrestrial disturbance, both of which can facilitate the establishment and spread of nonnative species at these sites [21]. The establishment of nonnative species is connected to disturbance through an increase of available resources, such as space, nutrients, and light [21, 22, 23, 24]. However, invasive species establishment also depends on the particular species and propagules introduced [25, 26, 27], and small seed mass, including seeds that can easily be accidentally dispersed, is one indicator of invasive species abundance [28]. With an increasingly global economy, *Industrialized Floras* are becoming a dominant, yet understudied, feature on the landscape [29, 30].

### Study at the Port of Savannah: An inventory of the *Industrialized Flora*

Port of Savannah operations are conducted by the Georgia Ports Authority [GPA; 31], which is a private-public partnership between the State of Georgia and private industry for the purpose of conducting and facilitating global trade. GPA oversees the import and export activities for the interchange of commodities to and from the region. In partnership with GPA, federal agencies such as the US Customs & Border Protection (USCBP) and the US Department of Agriculture (USDA), Animal-Plant Health Inspection Service (APHIS), Plant Pest Quarantine (PPQ), are tasked with the responsibility of serving as gatekeepers for the spectrum of activities associated with international trade at this port-of-entry. We partnered with GPA through their Client Relations Center to develop this study. We discovered that private industry, in this case GPA, possesses a vested interest for a multitude of benefits: (1) Evidence-based science to inform GPA practices and client relationships to improve and provide the most progressive and high-quality trade experience, (2) partnerships with research to streamline practices and facilities to improve rates of interceptions and reduce inadvertent biological contamination, and (3) reduction of client and GPA fiscal output due to biocontamination, including efforts to reduce frequency of fumigation and other control costs. Additionally, the government agencies are tasked with the protection of United States agricultural and agro-forestry interests, including conducting phytosanitary screenings and inspections to prevent the invasion of Federal Noxious Weeds [32].

The number of gatekeepers at these points-of-entry, however, are not nearly enough to keep pace with trade volumes, with the Port of Savannah seeing more than four million trade unit equivalents (TEUs; a standard shipping container measure) in 2017 alone [31]. Upon arrival at the seaport, the commodities within and on shipping containers are distributed by road and rail throughout the USA within 24 to 48 hours (Fig 2). Therefore, a combination of understaffed agricultural inspectors, massive trade volumes, and the swift transport of commodities across a complex network of roads and rails suggest that a relatively large portion of nonnative plant propagules are likely being moved from major seaports, inland, without interception or scientific awareness.

## Materials and methods

During project development, we met multiple times with GPA, the federal and state agencies involved, and university researchers on the ground at the GCT and defined our approach and a plan of action that met our shared aims to protect agricultural commodities and the industry in cooperation with USCBP and USDA, APHIS (Table 1). Ultimately, we discovered that partnerships between university and federal research aligned with the aims of the regulatory federal agencies and private industry, for a winning combination for all those involved (Fig 1). We assessed the *Industrialized Flora* across four major phenological timepoints at the GCT site to determine the baseline plant community at the container terminal.

Due to the high national security level required at a site where the initial entry of international goods and people occur, additional activities were necessary to obtain access to inventory the GCT on the Savannah River. This required a great deal of relationship-building, coordination, and permissions. Since this area had never been floristically inventoried since the GCT was constructed, we needed to assumed that Federal Noxious Weeds [32] may be present on-site and possibly collected; therefore, we applied for and was granted a USDA APHIS, PPQ 526 permit (#P526P-16-00812). Site inventory dates required coordination for permissions from GPA and GPA police, as well as USCBP supervisory escort for safety purposes and to reduce our impact on terminal activities. This included requesting and coordinating with GPA Police leadership to obtain permissions and support to allow us access into restricted (barb-wire fenced) greenspaces on GCT. For these highly-restricted areas, GPA Police provided us entry and was present during the entirety of our collection of those sites for all dates of survey. For university and research personnel that did not possess requisite Homeland Security clearance, background checks and visitor badging was required on the morning of each survey date prior to arriving on the GCT. A Supervisory Agriculture Specialist (MAK) from the USCBP for the Port of Savannah-area met us at one of the entry gates to ensure all badging and security attire met entry criteria, and gate security personnel were already alerted to our arrival from our coordination with GPA Police.

### Study site

At the Port of Savannah, the main container terminal is the GCT, located in Chatham County, Georgia, USA (32°07.3'N, 81°08.4'W). The GCT is the fourth-busiest container facility by volume, is greater than 485.5 ha in land area, and is the largest and busiest single-terminal container operation in the USA [31]. Up to 20,000 containers are moved daily at the terminal and in fiscal year 2015, 23.5 trillion kg of containerized cargo were moved, which was a 7.8% increase from 2014.

**Fig 3:**
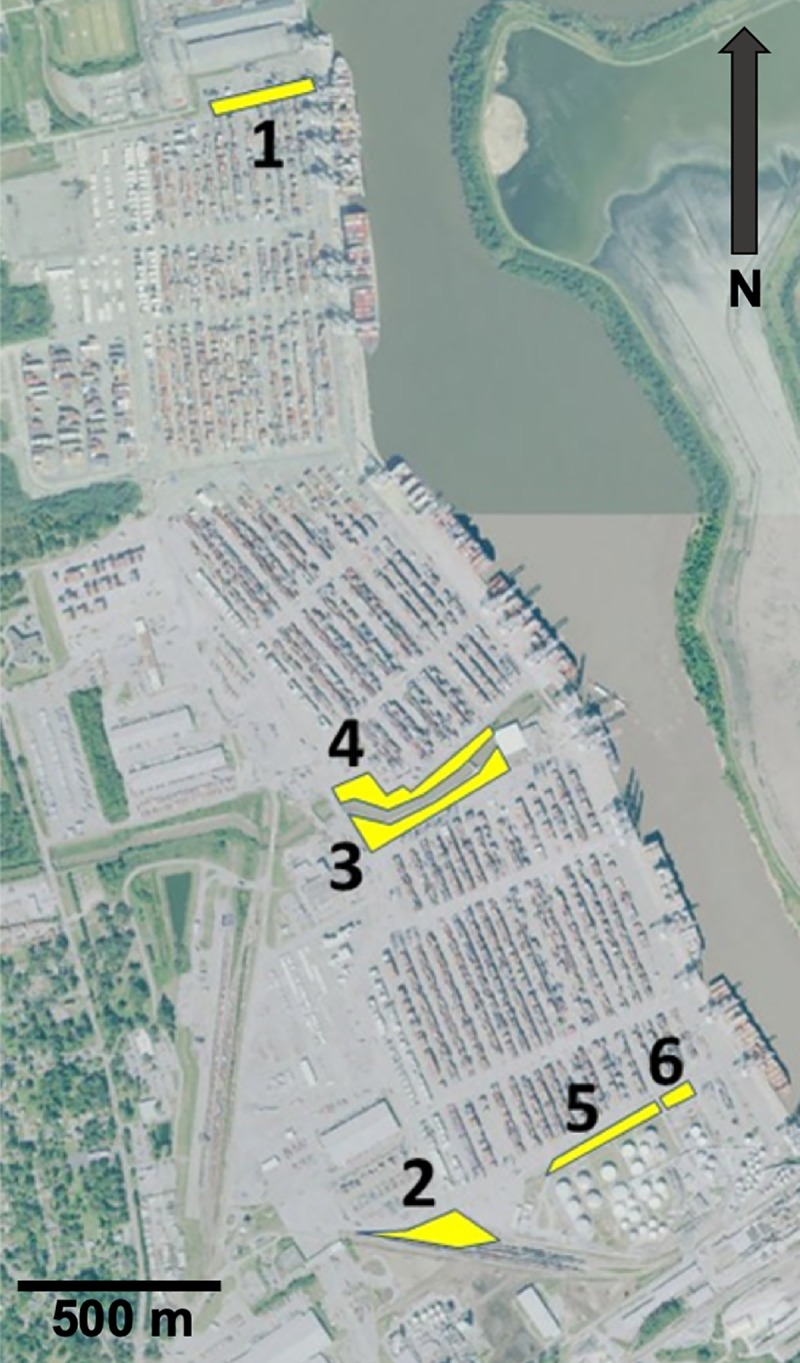
The Garden City Terminal (GCT) at the Port of Savannah, Georgia, USA. Yellow highlighted areas were inventoried. The base map from USGS National Map Viewer (http://viewer.nationalmap.gov/viewer/) is the USGS The National Map: Orthoimagery. Data refreshed April, 2019.

We identified six greenspaces on GCT (a high-security area) with the escort of GPA Police and USCBP inspectors, which totaled approximately 4.51 ha (or nearly 1% of the port property) (Fig 3). Area 1 is located at the northern edge of the GCT, Area 2 borders railroad tracks on the southwest edge of the property, Areas 3 and 4 border opposing sides of a small central channel for water runoff and are separated from the rest of the GCT by a chain-link fence, and Areas 5 and 6 border two sections of another small water runoff channel at the southeast end of the GCT (Fig 3).

### Floristic inventory

To assess the plant community at GCT, we performed four floristic surveys between August 2015 and February 2017 to capture as many of the plant species that grow on the limited greenspaces at the GCT. Surveys were conducted during daylight, with USCBP and GPA Police escorts, on 28 August 2015 (late summer), 19 May 2016 (late spring/early summer), 14 November 2016 (late fall/early winter), and 27 February 2017 (late winter/early spring). This periodical sampling approach allowed us to sample potentially different and diverse plant communities to best capture the plant species richness for the GCT [33].

During each survey, we collected all observable vascular plant species that were reproductive for the purpose of improving taxonomic identification from morphological characters. All vouchers were collected in duplicate and were identified utilizing classic taxonomic identification based on morphological characters. At sampling time, all specimens were pressed on-site. Each was assigned a unique collector identification number, and the area of GCT from which it was collected was described. Dried specimens, including duplicates, were sorted and sent to the Arkansas State University Herbarium (STAR) for morphological identification and deposition. Identified duplicates were sent to Columbus State University Herbarium (COLS; Table 1).

### Data management and statistical analysis

After we performed the surveys, floristic inventories conducted in Georgia or South Carolina were compiled for comparison to our inventory at the Port of Savannah. We primarily searched for the comparison floras by using *the FloraS of North America* database [34]. Additional floras were searched in the library of the University of Georgia Herbarium and through a web search on Google Scholar. In total, 30 other comparison floras were found; however, we selected a subset of studies as appropriate comparison floras if they were published in 1990 or more recently, if they covered an area less than the size of a county, and if the area of study was included in the manuscript. In all, 27 comparison floras were discovered that met our criteria (S1 Table).

All species reported in the studies were tabulated to build a comparison matrix that allowed us to analyze the studies based on the number and identity of the species reported in each study. We started with digital copies of each published manuscript (citations in S1 Table), and we used Tabula Version 1.2.1 (https://tabula.technology/) to turn species lists reported in each publication from PDF format into .csv for manual processing in Microsoft Excel (Microsoft Corporation, 2018. *Microsoft Excel*, Available at: https://office.microsoft.com/excel) and Google Sheets (https://www.google.com/sheets/about/). In many cases, there was a lot of additional information such as plant family name and habitat information in the raw conversion from PDF to a format usable in a spreadsheet software, and we used OpenRefine Version 3.3-beta (https://openrefine.org/) and manual effort in Microsoft Excel to clean the data, so that we ended with an alphabetical species list for each of the comparison floras.

We compared the number of species reported in each study to the number of taxa in our species lists generated in Excel (S1 Table). In all cases, we were within 5% of the reported value, and we deemed this appropriate given that Palmer and Richardson (2012) [35] found disagreement between the number of taxa reported in the abstract and the text or the abstract and the list of species as a common error in floristic studies. Since there have been a number of taxonomic nomenclatural changes from 1990 through the present, we standardized the nomenclature in each of the comparison floras using the Global Biodiversity Information Facility (GBIF) species matching tool at (https://www.gbif.org/tools/species-lookup). Some of the scientific names (77 of 12,484 = 0.6%) could not be reconciled with the GBIF species matching tool, and these names were excluded. Excluded species were relatively evenly distributed across the comparison floras with 8 of the floras (including ours) having no species excluded, and all others having fewer than 10 species excluded except for Comparison Flora 8, which had 15 species excluded (15 of 523 total reported species = 3%). In addition to species number and identity, we gathered information on the survey area (in hectares) and the number (and percent) of nonnative species from the text of each of the comparison floras.

The list of 12,484 species by comparison flora combination (S2 Table) was converted into a matrix (S3 Table) in RStudio (RStudio Team 2015—RStudio: Integrated Development for R. RStudio, Inc., Boston, MA URL http://www.rstudio.com/) using the provided code (SI Code 1). We referenced Zomlefer et al. (2013) [36] to include coefficients of conservatism (CofC), wetland indicator status (WIS), and native status of the species in our matrix. The matrix was then used in the following analyses: 1) the Species-Area Relationship (SAR) using the function: *SSarrhenius* (where, *S = kA*^*z*^; where *k*, is the constant based on the unit area; which in this case is hectares (ha). The slope of the line solved is indicated by the power function *z*, based on arithmetic axes, and where *S* is equal to the number of species.) in the R package *vegan* version 2.5–6 (https://cran.r-project.org), and 2) a Bray-Curtis dissimilarity matrix [37] generated for both CofC and WIS and then visualized using the *metaMDS* function [38, 39] in the R package *vegan*. Both the species-area relationship and nonmetric multidimensional scaling (NMDS) were conducted in R version 3.6.2 (R Core Team 2017). Frequencies of ordinal data from coefficient of conservatism and wetland status from the GCT survey (CF28) and 27 other comparative floras were calculated from the presence-absence species-by-site matrix (S3 Table). Frequency counts were then conducted and visualized in Microsoft Excel.

## Results

Our floristic inventory conducted at GCT resulted in 280 specimens (S1 Fig). From the collections, we identified a total of 174 species based on morphology (S1 Fig), representing 130 genera and 51 families (Table 2). More plant species were collected during the surveys in the most active growing seasons, and the percentage of new species encounters decreased with each subsequent sampling visit (S1 Fig; Table 2). Of 174 total species identified from the GCT surveys, 113 of those are native to the southeastern region of the USA, and 61 are nonnative (USDA PLANTS Database 2019; Table 2). Chatham County, Georgia, is a well-collected county with 1725 species and sub-specific taxa documented [40]. Still, we documented four state records, including two native (*Ipomoea nil* (L.) Roth and *Ludwigia bonariensis* (Micheli) H. Hara) and two introduced (*Crotalaria incana* L. and *Glandularia tenera* (Spreng.) Cabrera) species that have previously not been reported as growing in Georgia (Table 2) [40]. We also found 24 county records for species previously not documented in Chatham County, Georgia, of which 50% are native to the USA and 50% are nonnative (Table 2) [40]. No listed Federal Noxious Weeds were collected from our surveys on the GCT at the Port of Savannah.

**Table 2:** List of species comprising the *Industrialized Flora* at the Port of Savannah, Georgia, USA. Within the Native / introduced column, (SR) indicates a state record for a species that has not previously been reported as occurring within Georgia, and (CR) indicates a county record for a species that has not previously been reported in Chatham County, Georgia, according to the Biota of North America Program (BONAP) [40]. All species are represented by vouchers stored at Arkansas State University Herbarium (STAR) and Columbus State University Herbarium (COLS).

Plant family	Species name	Native / introduced	Number of surveys collected	Collected August 2015 (late summer)	Collected May 2016 (late spring / early summer)	Collected November 2016 (late fall / early winter)	Collected February 2017 (late winter / early spring)
**Alismataceae**	*Sagittaria lancifolia* L.	native	2	●	●		
**Altingiaceae**	*Liquidambar styraciflua* L.	native	1	●			
**Amaranthaceae**	*Alternanthera philoxeroides* (Mart.) Griseb.	introduced	1		●		
	*Amaranthus cannabinus* (L.) Sauer	native	1	●			
**Apiaceae**	*Chaerophyllum tainturieri* Hook. & Arn.	native	1				●
	*Cyclospermum leptophyllum* (Pers.) Sprague	introduced	1		●		
	*Eryngium aquaticum* L.	native	1	●			
	*Hydrocotyle umbellata* L.	native	1				●
	*Ptilimnium capillaceum* (Michx.) Raf.	native	1		●		
**Apocynaceae**	*Nerium oleander* L.	introduced (CR)	1		●		
**Aquifoliaceae**	*Ilex vomitoria* Aiton	native	1		●		
**Arecaceae**	*Sabal palmetto* (Walter) Lodd. ex Schult. & Schult. f.	native	1			●	
**Aspleniaceae**	*Asplenium platyneuron* (L.) Britton, Sterns & Poggenb.	native	1		●		
**Asteraceae**	*Ambrosia artemisiifolia* L.	native	3	●		●	●
	*Baccharis halimifolia* L.	native	2	●		●	
	*Bidens laevis* (L.) Britton, Sterns & Poggenb.	native	1			●	
	*Bidens pilosa* L.	introduced	3		●	●	●
	*Cirsium discolor* (Muhl. ex Willd.) Spreng.	native (CR)	1				●
	*Erechtites hieraciifolius* (L.) Raf. ex DC.	native	1	●			
	*Erigeron bonariensis* L.	introduced	2	●	●		
	*Erigeron canadensis* L.	native	1	●			
	*Erigeron strigosus* Muhl. ex Willd.	native	1		●		
	*Eupatorium capillifolium* (Lam.) Small ex Porter & Britton	native	1			●	
	*Gamochaeta pensylvanica* (Willd.) Cabrera	native	1		●		
	*Gamochaeta purpurea* (L.) Cabrera	native	1		●		
	*Helenium amarum* (Raf.) H. Rock	native	4	●	●	●	●
	*Heterotheca subaxillaris* (Lam.) Britton & Rusby	native	2	●			●
	*Hypochaeris glabra* L.	introduced	1				●
	*Lactuca canadensis* L.	native (CR)	2		●		●
	*Marshallia obovata* (Walter) Beadle & F. E. Boynton	native (CR)	1			●	
	*Packera glabella* (Poir.) C. Jeffrey	native	1				●
	*Pseudognaphalium obtusifolium* (L.) Hilliard & B. L. Burtt	native	1		●		
	*Pyrrhopappus carolinianus* (Walter) DC.	native	3	●	●		●
	*Pyrrhopappus pauciflorus* (D. Don) DC.	native (CR)	1		●		
	*Senecio vulgaris* L.	introduced (CR)	1				●
	*Solidago altissima* L.	native	1			●	
	*Solidago erecta* Banks ex Pursh	native (CR)	1			●	
	*Solidago leavenworthii* Torr. & A. Gray	native	1		●		
	*Sonchus oleraceus* L.	introduced	3		●	●	●
	*Symphyotrichum pilosum* (Willd.) G. L. Nesom	native	2		●	●	
	*Symphyotrichum puniceum* (L.) Á. Löve & D. Löve	native	1			●	
	*Symphyotrichum subulatum* (Michx. G. L. Nesom	native	1			●	
	*Vernonia altissima* Nutt.	native	1			●	
**Bignoniaceae**	*Campsis radicans* (L.) Seem.	native	2	●	●		
**Brassicaceae**	*Lepidium virginicum* L.	native	2		●		●
**Campanulaceae**	*Triodanis biflora* (Ruiz & Pav.) Greene	native (CR)	1				●
	*Wahlenbergia marginata* (Thunb.) A. DC.	introduced	2		●		●
**Caprifoliaceae**	*Lonicera japonica* Thunb.	introduced	3	●	●	●	
**Caryophyllaceae**	*Cerastium glomeratum* Thuill.	introduced	1				●
	*Silene antirrhina* L.	native	1				●
	*Spergularia marina* (L.) Besser	native	1				●
	*Stellaria media* (L.) Vill.	introduced	1				●
**Commelinaceae**	*Murdannia nudiflora* (L.) Brenan	introduced	1	●			
**Convolvulaceae**	*Ipomoea coccinea* L.	introduced (CR)	1			●	
	*Ipomoea nil* (L.) Roth	native (SR)	1			●	
	*Ipomoea trichocarpa* Elliott	native	2		●	●	
	*Jacquemontia tamnifolia* (L.) Griseb.	native	2	●		●	
**Cupressaceae**	*Juniperus virginiana* L.	native	2	●	●		
**Cyperaceae**	*Carex longii* Mack.	native	1		●		
	*Carex lupulina* Muhl. ex Willd.	native	1		●		
	*Cyperus compressus* L.	native	2	●		●	
	*Cyperus echinatus* (L.) Alph. Wood	native	1	●			
	*Cyperus iria* L.	introduced	2	●		●	
	*Cyperus planifolius* Rich.	native (CR)	1		●		
	*Cyperus strigosus* L.	native	1			●	
	*Cyperus surinamensis* Rottb.	native	4	●	●	●	●
	*Cyperus virens* Michx.	native	1		●		
	*Fimbristylis miliacea* (L.) Vahl	introduced	1			●	
**Euphorbiaceae**	*Acalypha gracilens* A. Gray	native	1		●		
	*Euphorbia maculata* L.	native	1	●			
	*Euphorbia nutans* Lag.	native	1	●			
	*Triadica sebifera* (L.) Small	introduced	2	●	●		
**Fabaceae**	*Alysicarpus ovalifolius* (Schum.) Leonard	introduced (CR)	1			●	
	*Chamaecrista nictitans* (L.) Moench	native	2	●		●	
	*Crotalaria incana* L.	introduced (SR)	1		●		
	*Crotalaria lanceolata* E. Mey.	introduced	1	●			
	*Macroptilium lathyroides* (L.) Urb.	introduced (CR)	1	●			
	*Medicago lupulina* L.	introduced	2		●	●	
	*Medicago polymorpha* L.	introduced	1				●
	*Melilotus albus* Medik.	introduced	3	●	●		●
	*Melilotus indicus* (L.) All.	introduced	1				●
	*Sesbania drummondii* (Rydb.) Cory	native	2	●	●		
	*Sesbania herbacea* (Mill.) McVaugh	native	1	●			
	*Strophostyles helvola* (L.) Elliott	native	1	●			
	*Strophostyles umbellata* (Willd.) Britton	native	1			●	
	*Trifolium arvense* L.	introduced (CR)	1		●		
	*Trifolium incarnatum* L.	introduced (CR)	1				●
	*Trifolium resupinatum* L.	introduced (CR)	1		●		
	*Vicia hirsuta* (L.) Gray	introduced	1				●
	*Vicia sativa* L.	introduced	1				●
	*Vigna luteola* (Jacq.) Benth.	native	2		●	●	
**Fagaceae**	*Quercus nigra* L.	native	1	●			
**Geraniaceae**	*Geranium carolinianum* L.	native	2			●	●
**Iridaceae**	*Sisyrinchium rosulatum* E. P. Bicknell	native	1		●		
**Juncaceae**	*Juncus diffusissimus* Buckley	native (CR)	1		●		
	*Juncus effusus* L.	native	1		●		
	*Juncus secundus* P. Beauv. ex Poir.	native (CR)	1		●		
	*Juncus validus* Coville	native	1		●		
**Lamiaceae**	*Lamium amplexicaule* L.	introduced	1				●
	*Scutellaria racemosa* Pers.	introduced	2		●		●
	*Stachys floridana* Shuttlew. ex Benth.	native	1			●	
**Lauraceae**	*Cinnamomum camphora* (L.) J. Presl	introduced	2	●			●
**Lygodiaceae**	*Lygodium japonicum* (Thunb.) Sw.	introduced	1		●		
**Malvaceae**	*Melochia corchorifolia* L.	introduced	1			●	
	*Sida rhombifolia* L.	native	2	●	●		
**Moraceae**	*Morus alba* L.	introduced	3	●	●		●
**Myricaceae**	*Morella cerifera* (L.) Small	native	2	●	●		
**Onagraceae**	*Ludwigia bonariensis* (Micheli) H. Hara	native (SR)	1		●		
	*Ludwigia decurrens* Walter	native	2		●	●	
	*Oenothera laciniata* Hill	native	2		●		●
**Orchidaceae**	*Spiranthes vernalis* Engelm. & A. Gray	native	1		●		
	*Zeuxine strateumatica* (L.) Schltr.	introduced (CR)	1				●
**Oxalidaceae**	*Oxalis corniculata* L.	native	1			●	
	*Oxalis dillenii* Jacq.	native	2		●		●
	*Oxalis violacea* L.	native (CR)	1				●
**Papaveraceae**	*Fumaria officinalis* L.	introduced	2		●		●
**Pinaceae**	*Pinus taeda* L.	native	2	●			●
**Plantaginaceae**	*Nuttallanthus canadensis* (L.) D.A. Sutton	native	1				●
	*Plantago lanceolata* L.	introduced	2	●	●		
	*Plantago major* L.	introduced (CR)	1		●		
	*Plantago virginica* L.	native	1		●		
	*Veronica agrestis* L.	introduced (CR)	1				●
**Poaceae**	*Agrostis hyemalis* (Walter) Britton, Sterns & Poggenb.	native	1		●		
	*Andropogon glomeratus* (Walter) Britton, Sterns & Poggenb.	native	1			●	
	*Andropogon virginicus* L.	native	1		●		
	*Bromus catharticus* Vahl	introduced	1		●		
	*Cenchrus echinatus* L.	native	2	●		●	
	*Cynodon dactylon* (L.) Pers.	introduced	1	●			
	*Dactyloctenium aegyptium* (L.) Willd.	introduced	1			●	
	*Digitaria ciliaris* (Retz.) Koeler	native	1	●			
	*Digitaria sanguinalis* (L.) Scop.	introduced	1			●	
	*Echinochloa colona* (L.) Link	introduced	1		●		
	*Eleusine indica* (L.) Gaertn.	introduced	2	●		●	
	*Eragrostis curvula* (Schrd.) Nees	introduced	1		●		
	*Eragrostis minor* Host	introduced (CR)	1	●			
	*Eragrostis secundiflora* J. Presl	native	1	●			
	*Lolium perenne* L.	introduced	2		●		●
	*Panicum scoparium* Lam.	native	1		●		
	*Paspalum notatum* Flüggé	native	2		●		●
	*Paspalum urvillei* Steud.	introduced	3	●	●		●
	*Phalaris caroliniana* Walter	native	1				●
	*Phragmites australis* (Cav.) Trin. ex Steud.	introduced	2	●	●		
	*Poa annua* L.	introduced	1				●
	*Setaria parviflora* (Poir.) M. Kerguelen	native	2	●	●		
	*Sorghum halepense* (L.) Pers.	introduced	2	●	●		
	*Sphenopholis obtusata* (Michx.) Scribn.	native	2		●		●
	*Sporobolus indicus* (L.) R. Br.	introduced	1	●			
**Polygonaceae**	*Persicaria hydropiperoides* (Michx.) Small	native	1		●		
	*Rumex hastatulus* Baldwin	native	1				●
	*Rumex verticillatus* L.	native	1		●		
**Pontederiaceae**	*Eichhornia crassipes* (Mart.) Solms	introduced	1			●	
	*Pontederia cordata* L.	native	1		●		
**Portulacaceae**	*Portulaca amilis* Speg.	introduced	1	●			
	*Portulaca pilosa* L.	native	1	●			
	*Portulaca smallii* P. Wilson	native (CR)	1		●		
**Primulaceae**	*Anagallis arvensis* L.	introduced	2		●		●
**Rosaceae**	*Rubus argutus* Link	native	1		●		
	*Rubus trivialis* Michx.	native	2	●			●
**Rubiaceae**	*Diodia virginiana* L.	native	2	●	●		
	*Galium aparine* L.	native	1				●
	*Galium tinctorium* L.	native	1		●		
	*Richardia scabra* L.	native	2	●		●	
**Salicaceae**	*Salix nigra* Marshall	native	1	●			
**Sapindaceae**	*Acer rubrum* L.	native	1	●			
**Smilacaceae**	*Smilax smallii* Morong	native	1				●
**Solanaceae**	*Physalis angulata* L.	native	1	●			
	*Solanum americanum* Mill.	native (CR)	2	●			●
**Tamaricaceae**	*Tamarix gallica* L.	introduced	1		●		
**Typhaceae**	*Typha domingensis* Pers.	native	1	●			
**Verbenaceae**	*Glandularia tenera* (Spreng.) Cabrera	introduced (SR)	4	●	●	●	●
	*Phyla nodiflora* (L.) Greene	native	2	●	●		
	*Verbena brasiliensis* Vell.	introduced	3	●	●	●	
**Vitaceae**	*Ampelopsis arborea* (L.) Koehne	native	1	●			
	*Parthenocissus quinquefolia* (L.) Planch.	native	1	●			

**Fig 4:**
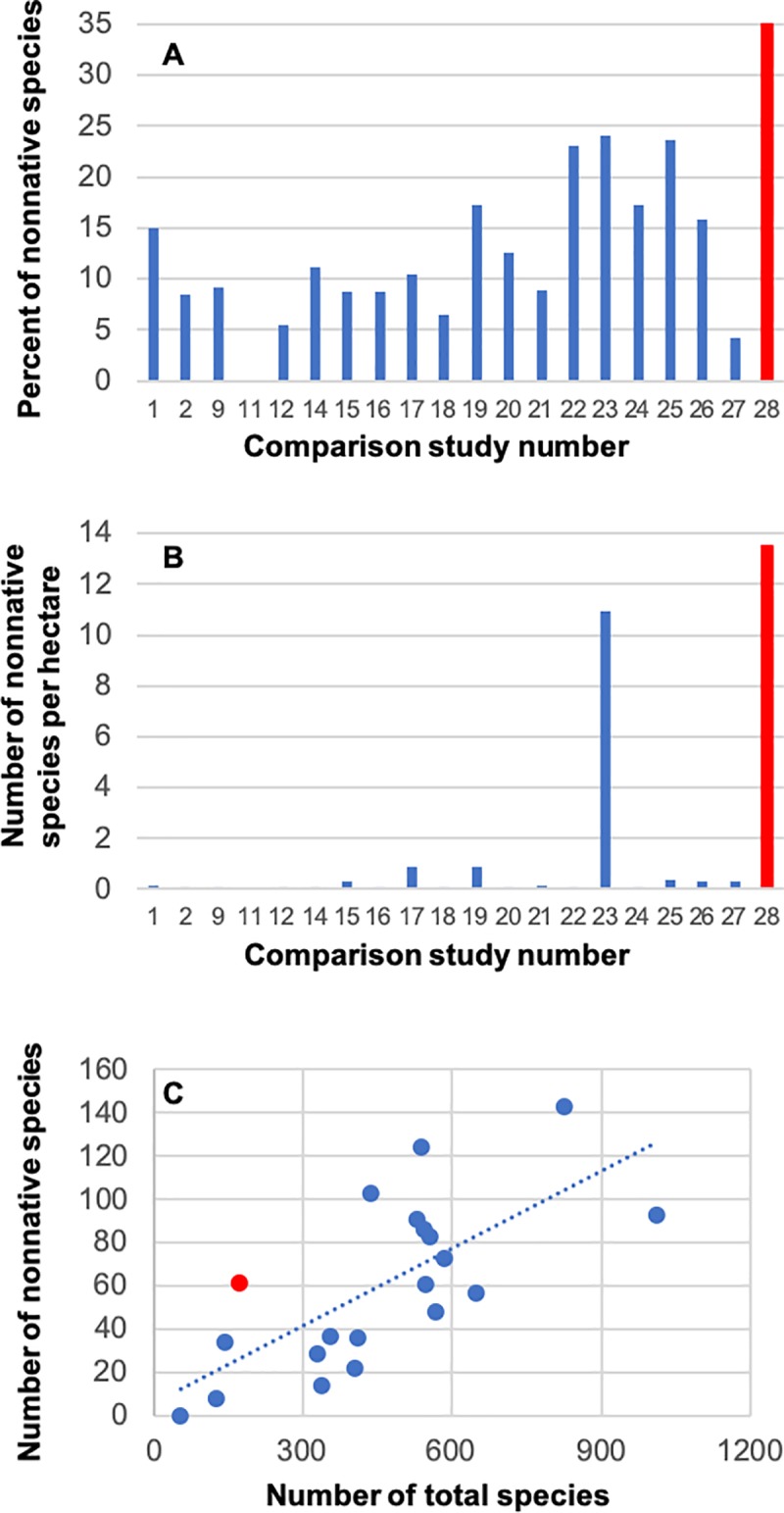
(A) Percent of nonnative species of the total species reported for 20 of the 28 comparison floras, (B) number of nonnative species per hectare of study area reported for 20 of the 28 comparison floras, and (C) scatterplot of number of nonnative species by number of total species reported for 20 of the 28 comparison floras. CF28 represents this study conducted at the Garden City Terminal, Port of Savannah, Georgia, USA, and bars and points are shown in red to highlight this study. Eight of the comparison floras did not directly report a number of nonnative species; therefore, they were excluded from this figure. Comparison floras 3–8, 10, and 13 are not shown.

When compared with the 27 other regional floras from Georgia and South Carolina, all other studies showed a lower percent of nonnative taxa (range = 0–24.1%; Fig 4A). The average percent of nonnative species was 13.3% for the floras when compared to 35.1% for our survey at GCT (Fig 4A). The average number of nonnative species per hectare (ha) was 1.41 for the comparison floras, whereas for the GCT, we recorded 13.5 nonnative species per hectare (Fig 4B). In the comparison floras, there is an increase in the number of nonnative species with an increase in the number of species overall, but CF28 (our study) shows a high number of nonnative species (far above the trendline) for the few species collected overall (Fig 4C).

**Fig 5:**
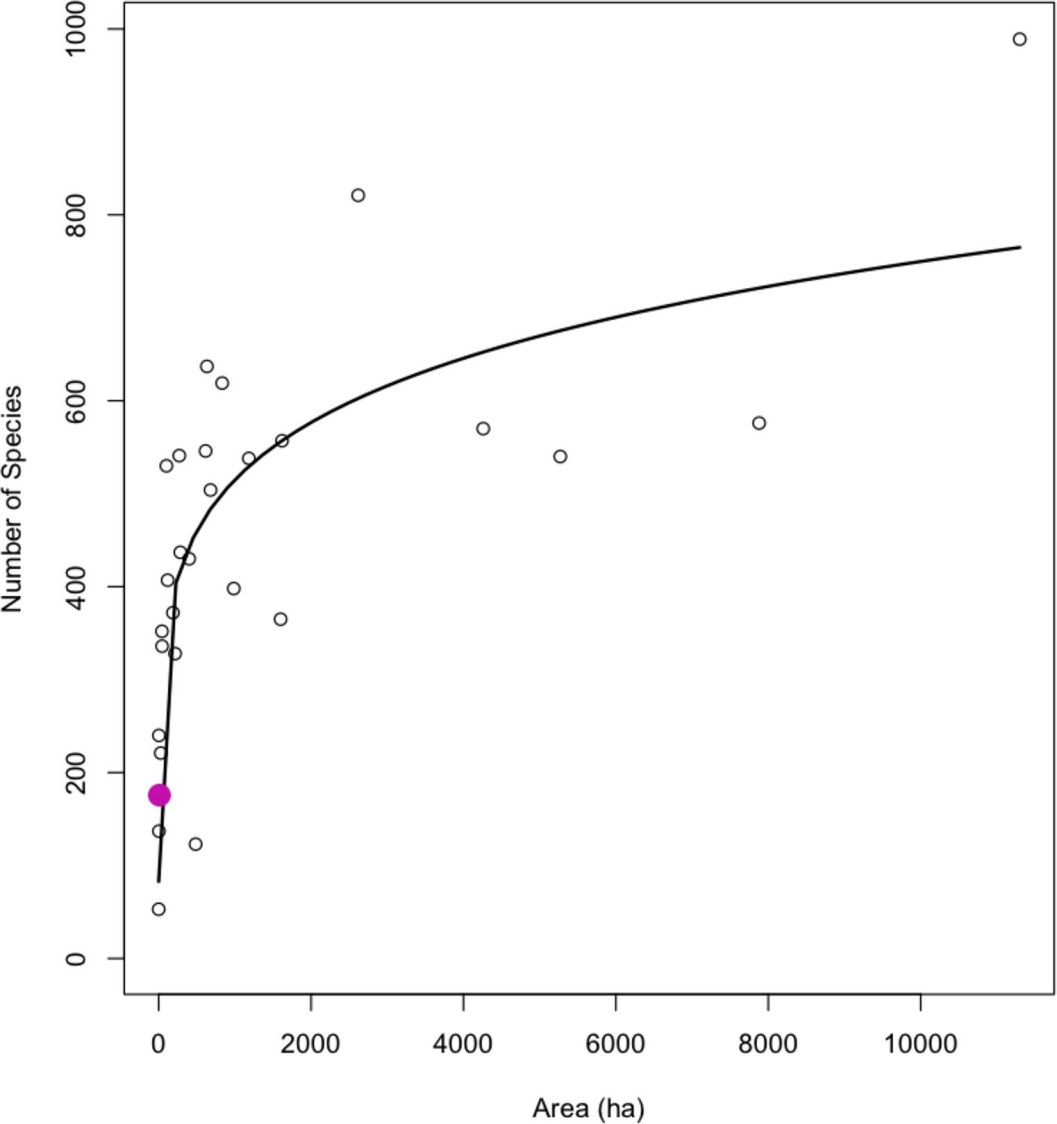
Species-area relationship of all 28 comparison floras (CF1 through CF28, labeled; S1 Table). The Garden City Terminal survey at the Port of Savanah (CF28) is the pink circle, and all 27 other comparison floras as open circles.

The species-area relationship among the 28 comparison floras as calculated by the species presence matrix (S3 Table), resulted in a species-area relationship (Fig 5; derived from *S = kA*^*z*^) where the constant based on the unit area, *k* = 166.8768 (ha), and *z* = 0.1631, the slope of the line from all 28 comparative surveys. The GCT survey (CF28) did not deviate from the species area-relationship based on this power-function (Fig 5). What we observed among the comparison floras (N = 28; S1 Table) was that as area (ha) increased, so did the number of species, as expected; however, deviation from the curve was observed to increase as area increased within our dataset of comparative floras. The survey at the GCT (CF28; pink dot, Fig 5) fell on the species-area relationship curve, in line with most other comparison floras (CFs), indicating our survey was comprehensive and comparable to other regional floras (S1 Table). The far outlier in Fig 5 (top right-hand corner), is CF9 (S1 Table), where high relative area resulted in a very high number of plant species collected, deviating more from other floras.

**Fig 6:**
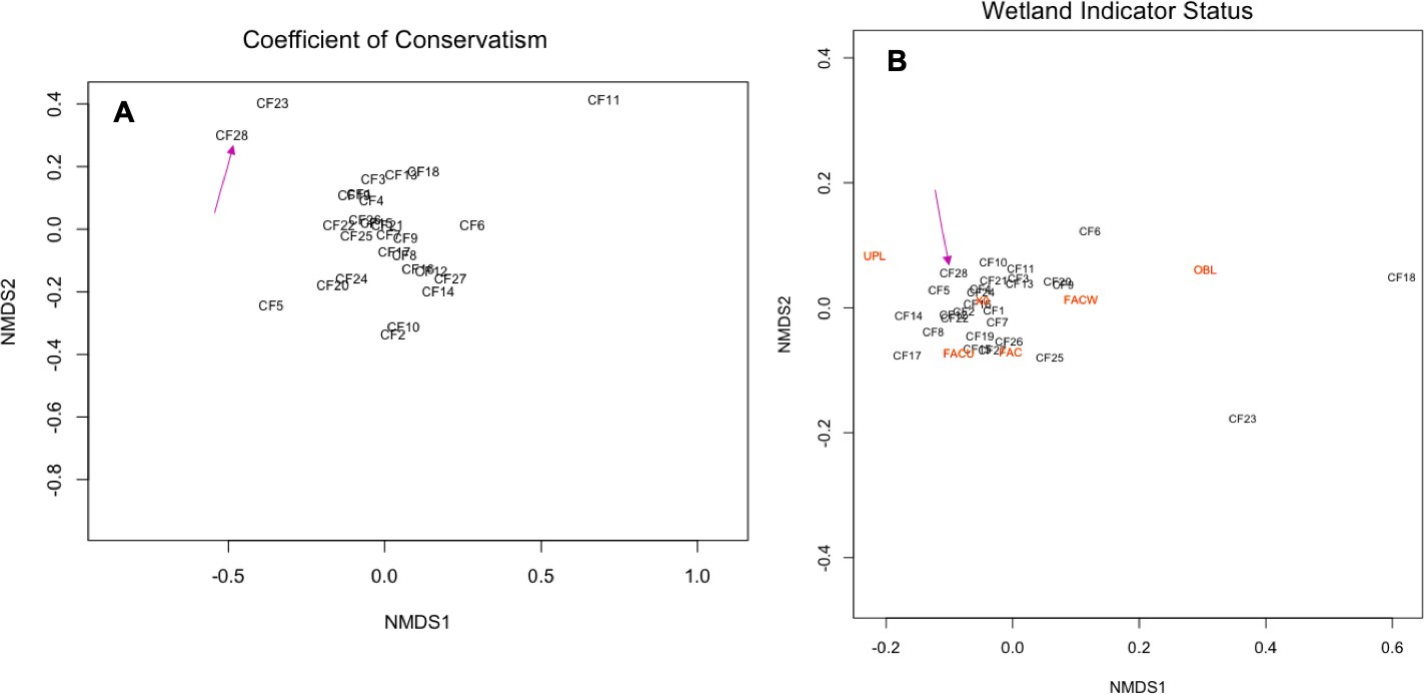
Nonmetric multidimensional scaling (NMDS) of (A) Coefficients of Conservatism (ranges from 0 to 10 [36]) frequency values, and (B) wetland indicator status frequency of each comparison flora, where the orange font indicates each category: facultative (FAC), facultative upland (FACU), facultative wetland (FACW), obligate wetland (OBL), and upland (UPL); NA indicates that wetland indicator status was not provided [36]). CF28 is pointed out with a pink arrow in each figure panel.

Bray-Curtis dissimilarity among comparison flora sites (CF1 through 28) were visualized using nonmetric multidimensional scaling (NMDS) of coefficient of conservatism values (Fig 6A) and wetland indicator statuses (Fig 6B). Mean frequencies of coefficient of conservatism ranged from 2.27 for CF28, our flora at the GCT, to 4.84 from CF11. All comparison floras (CF1 through 27; Table 3) resulted in coefficient of conservatism mean frequencies of >2.50 (Fig 6A, Table 3). Frequency of wetland statuses for all CFs (summarized in Table 3; visualized in Fig 6B) shows that the GCT flora (CF 28) was not outside the cloud of points when compared to the 27 other comparison floras.

**Table 3:** Summary of mean frequencies of plant species from each comparison flora assigned to a coefficient of conservatism ranking (values 0 to 10), and frequency of plant species of each flora for wetland indicator status. For coefficients of conservatism a zero (0) value represents an invasive species, a one (1) represents nonnative species, a two (2) is a non-conservative native species that is not specific to a habitat type [often considered weedy], and increasing numbers (to 10) represent species with a narrowing range of ecological tolerances and a decreasing ability to tolerate disturbance. These values follow Zomlefer et al. (2013) [36]. An NA value for wetland indicator status means that no wetland status was applied the species; wetland indicator statuses are facultative (FAC), facultative upland (FACU), facultative wetland (FACW), obligate wetland (OBL), and upland (UPL) [36].

	Coefficient of Conservatism	Frequency of Wetland Indicator Status for each inventory site					
Site	Mean Frequency	NA*	FAC	FACU	FACW	OBL	UPL
**CF1**	3.57	163	98	149	74	52	9
**CF2**	4.28	193	89	153	72	37	12
**CF3**	3.67	118	58	76	62	50	7
**CF4**	3.68	198	101	154	87	63	15
**CF5**	2.82	53	43	73	27	18	6
**CF6**	4.31	69	31	26	65	44	4
**CF7**	3.90	193	95	143	87	43	8
**CF8**	4.04	174	97	141	61	16	12
**CF9**	3.97	291	163	171	188	163	12
**CF10**	4.34	140	59	95	69	53	11
**CF11**	4.83	16	7	10	10	6	1
**CF12**	4.37	129	69	111	55	20	10
**CF13**	3.86	119	64	66	67	41	7
**CF14**	4.36	198	95	147	56	23	20
**CF15**	3.67	127	95	98	61	18	7
**CF16**	4.26	210	110	159	92	50	14
**CF17**	3.84	126	84	89	35	7	10
**CF18**	4.07	15	13	3	30	61	0
**CF19**	3.46	164	110	145	68	34	8
**CF20**	3.57	173	101	96	89	108	8
**CF21**	3.86	117	50	75	40	39	6
**CF22**	3.26	195	95	140	64	31	12
**CF23**	2.78	16	26	27	26	41	0
**CF24**	3.75	258	153	192	115	82	20
**CF25**	3.47	107	95	111	75	45	3
**CF26**	3.55	151	122	136	83	41	7
**CF27**	4.57	87	77	88	58	19	6
**CF28**	2.27	42	38	48	21	20	5

*NA = denotes no Wetland Indicator Status assigned for the species

**Fig 7:**
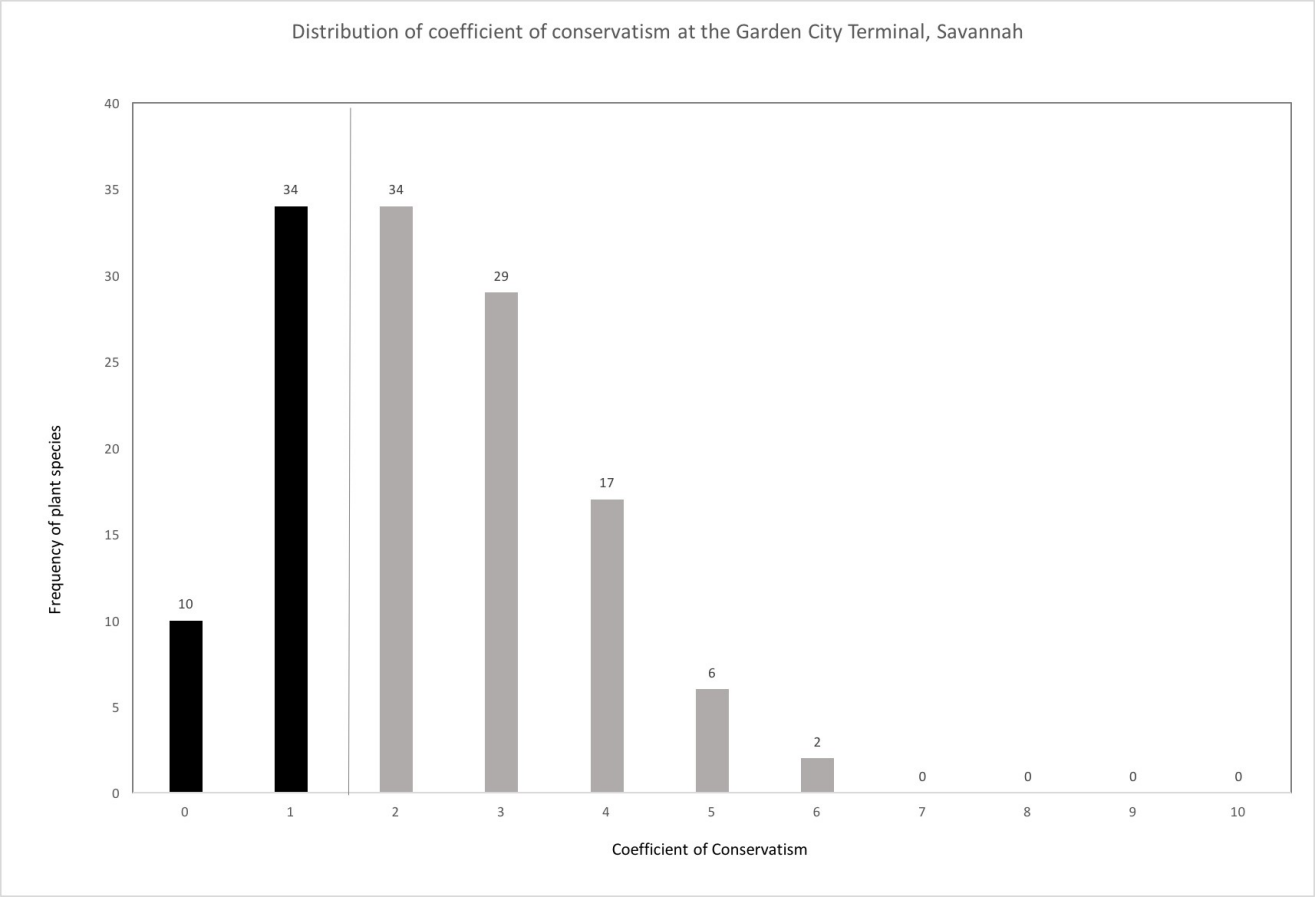
Distribution of the coefficients of conservatism of the species collected at the Garden City Terminal, Port of Savannah, Georgia, USA. Coefficients with a value of 0 or 1 represent nonnative species and those with a value of 2 or higher represent native species. Of the 174 species found at GCT, 42 did not have Coefficient of Conservatism values provided [36].

The main result from coefficients of conservatism across the comparison floras was that the GCT plant species distribution had a very high number of nonnative species (Fig 4A; Table 2; Table 3). Ten plant species were assigned a rank 0 (*where 0 = nonnative invasive*), followed by 34 species assigned a rank of 1 (*where 1 = relatively benign*, *nonnative species*; Fig 7) [36]. Thirty-four plant species were assigned to a rank of 2 (*where 2 = native*, *but restricted to areas of human disturbance*); no plant species at the GCT were assigned coefficients of conservatism 7 to 10 [36]. The general frequency of plant species collected from the GCT resulted in the highest number of invasive (0) and nonnative (1) species, with decreasing observations as the coefficient of conservatism increased (Fig 7), indicating that the GCT is comparatively a hot-spot of invasive and nonnative plant species that persist in anthropogenically disturbed sites.

## Discussion

The observed patterns between our study and the other comparison floras in the immediate region (1I Table) is to be expected considering the rapidity of biotic homogenization, due to anthropogenic activities [41] associated with a high-volume, high-traffic, and highly disturbed international trade hub. The large number of invasive and nonnative species [36] at the GCT relative to other surveys is consistent with our expectation that the shipping port is a unique site relative to the regional floristic research emphasis of the recent past (Table 3). The high proportion of nonnative species could increase over time, considering constant and increasing anthropogenic disturbance as well as nonnative propagule pressure of flora and fauna. Despite the GCT flora (CF28) emerging as a hot-spot of invasive and nonnative plant richness (Fig 7, Table 3), our flora did not deviate from the dataset of 28 total comparison floras (S1 Table) based on the species-area relationship (Fig 5). This suggests that our collection and inventory of vascular plants at the Port of Savannah was comprehensive and well-executed, making comparisons with other regional floras appropriate.

All comparison floras were conducted in natural habitats or areas of conservation concern, and we found that our *Industrialized Flora* from the GCT at the Port of Savannah shows a remarkable departure in invasive and nonnative taxa from what botanists generally inventory. It is expected that comparing our *Industrialized Flora* to a natural area or conservation area flora would result in substantial differences in species identities within the assemblage. Still, an overarching theme of our research is that there were no other similar urban or industrial comparison floras in Georgia and South Carolina for which we could include with our analyses. We advocate for more research at industrial sites and ports-of-entry so a vouchered knowledge-base of successful species introduction can be further documented.

The port only consists of small greenspaces (Fig 3), and it harbors only a small number of species, but it has a uniquely high number of nonnative and invasive species (Table 3, Fig 4A, Fig 4B, Fig 7). These patterns suggest that there is a combination of mechanisms at the shipping port to promote an unusually high number of nonnative species. Moreover, finding two nonnative species not previously recorded in Georgia, USA, and 12 nonnative species not previously recorded in Chatham County, Georgia, USA, underscores the novelty of introductions at international points-of-entry. It is unclear what the exact mechanisms are for the nonnative species hub at the GCT, but it could include multiple, non-mutually exclusive factors such as disturbance, propagule pressure, location, time, and/or climate. To further understand the relation between international trade destination and nonnative plant richness, additional assessments of *Industrialized Floras* need to be conducted at regional and global scales.

Not only are nonnative plants a concern, but animal species representing *Industrialized Faunas* also may insidiously establish at ports-of-entry. One of the best-known, most impactful recent examples is the introduction of emerald ash borer (EAB; *Agrilus planipennis* Fairmaire) in Detroit, Michigan, USA, likely in contaminated packing materials of commodity shipments [42, 43]. Ballast water exchange at freshwater and marine ports has resulted in the intercontinental exchange of many species, resulting in some very damaging invasive species such as zebra mussel (*Dreissena polymorpha* [Pallas]) and European green crab (*Carcinus maenas* L.) [44, 45, 46]. Therefore, not only *Industrialized Floras* need to be conducted and inventoried more often and at more sites, but an understanding of the *Industrialized Fauna* is a necessary preventative measure to better intercept nonnative hitchhikers and propagules using our suggested approach.

Our framework (Fig 1) aims to reduce the number of nonnative, invasive species of all taxa from being exchanged across nations. The framework to engage with private industry (Fig 1) is applicable to all taxa, and multiple inventories are needed by teams of scientists that can collaborate to accomplish the daunting task of reducing the introduction rates of nonnative species. We advocate for a more economically and ecologically advantageous pursuit in prevention (a 1:100 economic return) and early-detection/eradication (a 1:25 economic return) as compared to asset-based containment, which is employed when a species has already become established, widespread, and demonstrates a substantial negative impact (a >1:1–5 economic return) [47].

## Conclusions

We found that, in general, most botanical inventories avoid *Industrialized Floras* (S1 Table), including those located at major sites of international trade of large-volume commodities and human activity. One possible reason for this avoidance could be that heavy industry seems to be the antithesis of people’s perception of “fieldwork” and natural resources management. Rather, we found that industrial sites are of high value for the purpose of monitoring and studying nonnative species since these sites serve as initial points-of-entry and areas for nonnative species establishment. Another reason why *Industrialized Floras* are understudied is that security at these sites may represent a real or perceived barrier to researchers and botanists. Developing relationships among academic scientists, government agencies, and the private-sector, such as heavy industries, is key to having successful and long-term partnerships so that environmental and public needs are adequately and correspondingly addressed (Fig 1). Presently, successful plant invasions may be cryptically occurring due to a lack of broad-scale partnerships between research, governmental agencies, and private industry. Furthermore, the lack of inspectors and “boots-on-the-ground” at these major, international points-of-entry poses a significant risk to the national agriculture economy and biosecurity. We make the call to our colleagues and partners that we must urgently initiate more partnerships with industries of the private sector to further investigate the species composition of *Industrialized Floras* in order to stop the introduction and establishment of nonnative species at significant points-of-entry, such as seaports.

## Supporting information

S1 Table List of regional comparison floras included in our analyses to determine the uniqueness of the flora at the Garden City Terminal, Port of Savannah, Georgia, USA. (DOCX)

S2 Table Species identities from the 28 comparison floras with scientific names reconciled with the GBIF species matching tool. The CF Study Number column represents the comparison flora number from S1 Table.
(XLSX)

S3 Table Comparison flora species matrix. The GBIF-reconciled-name-with authority column contains a list of all species found throughout the 28 comparison floras. The columns numbered CF1-CF28 have a 0 in a cell for which a species was not found in the study and a 1 in a cell for which a species was found in the study. The CF Study Number column represents the comparison flora number from S1 Table.
(XLSX)

S1 Code Code used in R-studio to create S3 Table. (DOCX)

S1 Fig Number of species collected during each floristic survey. In blue is the number of new species collected on each subsequent survey date, demonstrating a decrease in novel encounters with increasing sampling effort. Purple bars represent the total number of species collected during the specific survey date, in which lower numbers in November 2016 and February 2017 are consistent with expected phenology for this region. In orange is the total accumulated species across the survey dates.
(DOCX)
