# Supplementary material for: An initial industrial flora: A framework for botanical research in cooperation with industry for biodiversity conservation
Source: PLoS One. 2020 Apr 1;15(4):e0230729. doi: 10.1371/journal.pone.0230729 (PMC7112212; doi:10.1371/journal.pone.0230729)
Supplement: S1 Table — (DOCX) [file pone.0230729.s001.docx]

**Supplementary Information**

**Supplementary Information Table 1.** List of regional comparison floras included in our analyses to determine the uniqueness of the flora at the Garden City Terminal, Port of Savannah, Georgia, USA.

| **Comparison flora study number** | **Study citation** | **Study area (ha)** | **Number of species reported in study** | **Number of nonnative species reported in study** | **Number of taxa in our compiled list (including infraspecific taxa)** | **Number of taxa removed because names not reconciled** | **Number of species total included in analyses after GBIF reconciliation** |
| --- | --- | --- | --- | --- | --- | --- | --- |
| **1** | Howel, C. L. 1991. Floristics of two state parks in the Piedmont of Georgia: Indian Springs and High Falls. Castanea 56: 38-50 | 615.2 | 556 | 83 | 553 | 0 | 545 |
| **2** | Tobe, J. D., J. E. III Fairey,, and L. L. Gaddy. 1992. Vascular flora of the Chauga River Gorge, Oconee County, South Carolina. Castanea 57: 77-109 | 1619 | 568 | 48 | 566 | 2 | 556 |
| **3** | Toole, M.A. Jr. 1992. A floristic study and vegetation analysis of the southern portion of the George L. Smith State Park in Emanuel County, Georgia, USA. Thesis (Georgai Southern University). | 187 | 374 | Not reported | 377 | 1 | 371 |
| **4** | Seward, J. E. 1993. Floristic survey of the Apalachee River corridor. Thesis (University of Georgia). | 833 | 591 | Not reported | 633 | 8 | 618 |
| **5** | Stalter, R. and E. E. Lamont. 1993. The vascular flora of Fort Sumter and Fort Moultrie, South Carolina, one year after Hurricane Hugo. Castanea 58: 141-152. | 27 | 223 | Not reported | 222 | 0 | 220 |
| **6** | Helton, R. C. 1995. A floristic study and vegetation analysis of a floristically diverse powerline right-of-way in Tattnall County, Georgia. Thesis (Georgia Southern University) | 2.95 | 258 | Not reported | 247 | 6 | 239 |
| **7** | Kennemore, D. E. Jr. 1995. Floristics of the Kings Mountain National Military Park and the Kings Mountain State Park. Thesis (University of South Carolina). | 4259 | 586 | Not reported | 594 | 9 | 569 |
| **8** | Milsted, D. L. 1997. Vascular flora of the Warwoman Wildlife Management Area (Rabun County, Georgia). Thesis (University of Georgia). | 6880 | 523 | Not reported | 523 | 15 | 503 |
| **9** | Drew, M. B., L. K. Kirkman, and A. K.. Gholson Jr. 1998. The vascular flora of Ichauway, Baker County, Georgia: a remnant longleaf pine/wiregrass ecosystem. Castanea 63: 1-24. | 11300 | 1013 | 93 | 1011 | 5 | 988 |
| **10** | Noel, W. G., 1998. Vascular flora of the Tallulah Gorge, Rabun and Habersham Counties, Georgia. Thesis (Clemson University). | 400 | 460 | Not reported | 446 | 7 | 429 |
| **11** | Hill, S. R. 1999. The relict flora of ice ponds in South Carolina. Castanea 64: 14-22. | 0.01365 | 53 | 0 | 53 | 1 | 52 |
| **12** | Waldrop, L. 2001. A floristic study of the Cane Creek watershed of the Jocassee Gorges property, Oconee and Pickens Counties, South Carolina. Thesis (Clemson University). | 985 | 403 | 22 | 402 | 1 | 397 |
| **13** | Luber, H. H. 2002. Floristic inventory of an Altamaha River floodplain area. Thesis (University of Georgia). | 1600 | 375 | Not reported | 374 | 1 | 364 |
| **14** | Moore, J. A. 2002. The vascular flora of Grassy Mountain, Murray County, Georgia. Thesis (University of Georgia). | 5270 | 548 | 61 | 556 | 1 | 539 |
| **15** | Schmidt, J. M. and J. A. Barnwell. A flora of the Rock Hill Blackjacks Heritage Preserve, York County, South Carolina. Castanea 69: 247-279. | 117.3 | 410 | 36 | 412 | 3 | 406 |
| **16** | Kruse, L. M. 2003. Vascular flora of the Upper Etowah River watershed, Georgia. Thesis (University of Georgia). | 633 | 649 | 57 | 661 | 5 | 636 |
| **17*** | Echols, S. L. 2007. Vascular flora of the remnant blackland prairies and associated vegetation of Georgia. Thesis (University of Georgia). | 43 | 354 | 37 | 352 | 0 | 351 |
| **18** | Stalter, R., J. Baden, and D. Kincaid. 2007. The vascular flora of three abandoned rice fields, Georgetown, South Carolina: a 39 year comparison. Journal of the Botanical Research Institute of Texas 1: 665-677. | 484.8 | 124 | 8 | 124 | 2 | 122 |
| **19** | Horn, C. N. 2008. A vascular flora of Lynch's Woods Park, Newberry County, South Carolina. Castanea 73: 111-122. | 101.2 | 528 | 91 | 530 | 0 | 529 |
| **20** | Zomlefer, W. B., D. E. Giannasi, K. A. Bettinger, S. L. Echols, and L. M. Kruse. 2008. Vascular plant survey of Cumberland Island National Seashore, Camden County, Georgia. Castanea 73: 251-282. | 7880 | 583 | 73 | 584 | 4 | 575 |
| **21** | Jenkins, R. A. and P. D. McMillan. 2009. Vascular flora of Sandhill Research and Education Center, Richland County, South Carolina. Castanea 74: 168-180. | 215 | 328 | 29 | 329 | 1 | 327 |
| **22** | Zomlefer, W. B., D. E. Giannasi, and S. L. Echols. 2010. Vascular plant flora of Kennesaw Mountain National Battlefield Park, Cobb County, Georgia. Southeastern Naturalist 9: 129-164. | 1183 | 538 | 124 | 543 | 1 | 537 |
| **23** | White, S. A., M. D. Taylor, and D. Z. Damrel. 2012. Floral colonization of a free-water surface constructed wetland system in Grady County, Georgia. Castanea 77: 159-171. | 3.1 | 141 | 34 | 139 | 2 | 136 |
| **24** | Zomlefer, W. B., D. E. Giannasi, A. Reynolds, and K. Heiman. 2012. Vascular Plant Flora of Chattahoochee River National Recreation Area, a Conservation Corridor from the Buford Dam to Atlanta, Georgia. Rhodora 114: 50-102. | 2618 | 827 | 143 | 830 | 2 | 820 |
| **25** | Zomlefer, W. B., D. E. Giannasi, J. B. Nelson, and L. L. Gaddy. A baseline vascular plant survey for Ocmulgee National Monument, Bibb County, Macon, Georgia. Journal of the Botanical Research Institute of Texas 7: 453-473. | 283.9 | 436 | 103 | 436 | 0 | 436 |
| **26**** | Sewell, S. Y. S. 2013. Floristic inventory and quality of Piedmont Gabbro Upland Depression Forests in Jasper County, Georgia. Thesis (University of Georgia). | 270 | 543 | 86 | 553 | 0 | 540 |
| **27** | Lynch, P. S. and W. B. Zomlefer. 2016. Vascular Plant Flora of the South Atlantic Coastal Plain Limestone Forest: A Globally Imperiled Association Endemic to Central Georgia. Southeastern Naturalist 15: 331-345. | 44.67 | 336 | 14 | 338 | 0 | 335 |
| **28** | Lucardi, R. D., C. E. Cunard, S. C. Hughes, K. S. Burgess, J. N. Reed, S. J. Worthy, L. W. Whitehurst, and T. D. Marsico. An initial industrial flora: a framework for botanical research in cooperation with industry for biodiversity conservation. PLoS One. | 4.51 | 174 | 44 | 174 | 0 | 174 |

*Excluded from our study was the peer-reviewed publication based on Echols 2007 MS thesis, which would represent redundant data in our dataset. Echols, S. L. and W. B. Zomlefer. 2010. Vascular plant flora of the remnant Blackland Prairies in Oaky Woods Wildlife Management Area, Houston County, Georgia. Castanea 75: 78-100.

**Excluded from our study was the peer-reviewed publication based on Sewell 2013 MS thesis, which would represent redundant data in our dataset. Sewell, S. Y. S and W. B. Zomlefer. 2014. Floristics of Piedmont Gabbro Upland Depression Forests in Jasper County, Georgia. Castanea 79: 195-220.
