# Supplementary material for: An initial industrial flora: A framework for botanical research in cooperation with industry for biodiversity conservation
Source: PLoS One. 2020 Apr 1;15(4):e0230729. doi: 10.1371/journal.pone.0230729 (PMC7112212; doi:10.1371/journal.pone.0230729)
Supplement: S1 Code — (DOCX) [file pone.0230729.s004.docx]

**Supplementary Information Code 1.**  Code used in R-Studio to create Supplementary Information Table 3.

#load library

library(tidyverse)

#read in comparison flora data from csv

data <- read_csv("All names compiled from all CF studies_77 rows removed lack GBIF reconcile_2 colums_for Erica_20200113.csv")

#discard duplicate rows (same taxon from the same study, due to synonyms) and spread data

#so that each study is represented as its own column vs. values in a single column

data_spread <- data %>%

distinct() %>%

mutate(present = 1) %>%

spread(key = "CF Study Number", value = "present")

#sum the number of unique taxa recorded per study and save as CSV file, for comparison with manuscripts

taxa_per_study <- data_spread %>%

select(-`GBIF-reconciled-name-with-authority`) %>%

colSums(na.rm = TRUE) %>%

bind_rows()

write_csv(taxa_per_study, "taxa_per_study.csv")

#read in C of C and wetalnd status data

data_CofCwetland <- read_csv("Zomlefer et al. 2013_CoC for GA_supplemental table_for Erica_20200113.csv")

data_CofCwetland <- data_CofCwetland %>% filter(!is.na(`GBIF_reconciled_with_authority`))

#join nativity status

data_spread_joined <- data_spread %>%

left_join(data_CofCwetland,

by = c("GBIF-reconciled-name-with-authority" = "GBIF_reconciled_with_authority")) %>%

select(`GBIF-reconciled-name-with-authority`, Nativity, `C of C`, AGCP, everything()) %>%

arrange(`GBIF-reconciled-name-with-authority`)

#save data_spread

write_csv(data_spread_joined, "all-names-compiled-from-all-CF-studies_spread.csv")
