# Supplementary material for: An initial industrial flora: A framework for botanical research in cooperation with industry for biodiversity conservation
Source: PLoS One. 2020 Apr 1;15(4):e0230729. doi: 10.1371/journal.pone.0230729 (PMC7112212; doi:10.1371/journal.pone.0230729)
Supplement: S1 Fig — In blue is the number of new species collected on each subsequent survey date, demonstrating a decrease in novel encounters with increasing sampling effort. Purple bars represent the total number of species collected during the specific survey date, in which lower numbers in November 2016 and February 2017 are consistent with expected phenology for this region. In orange is the total accumulated species across the survey dates. (DOCX) [file pone.0230729.s005.docx]

**Supplementary Information Figure 1.** Number of species collected during each floristic survey. In blue is the number of new species collected on each subsequent survey date, demonstrating a decrease in novel encounters with increasing sampling effort. Purple bars represent the total number of species collected during the specific survey date, in which lower numbers in November 2016 and February 2017 are consistent with expected phenology for this region. In orange is the total accumulated species across the survey dates.

**
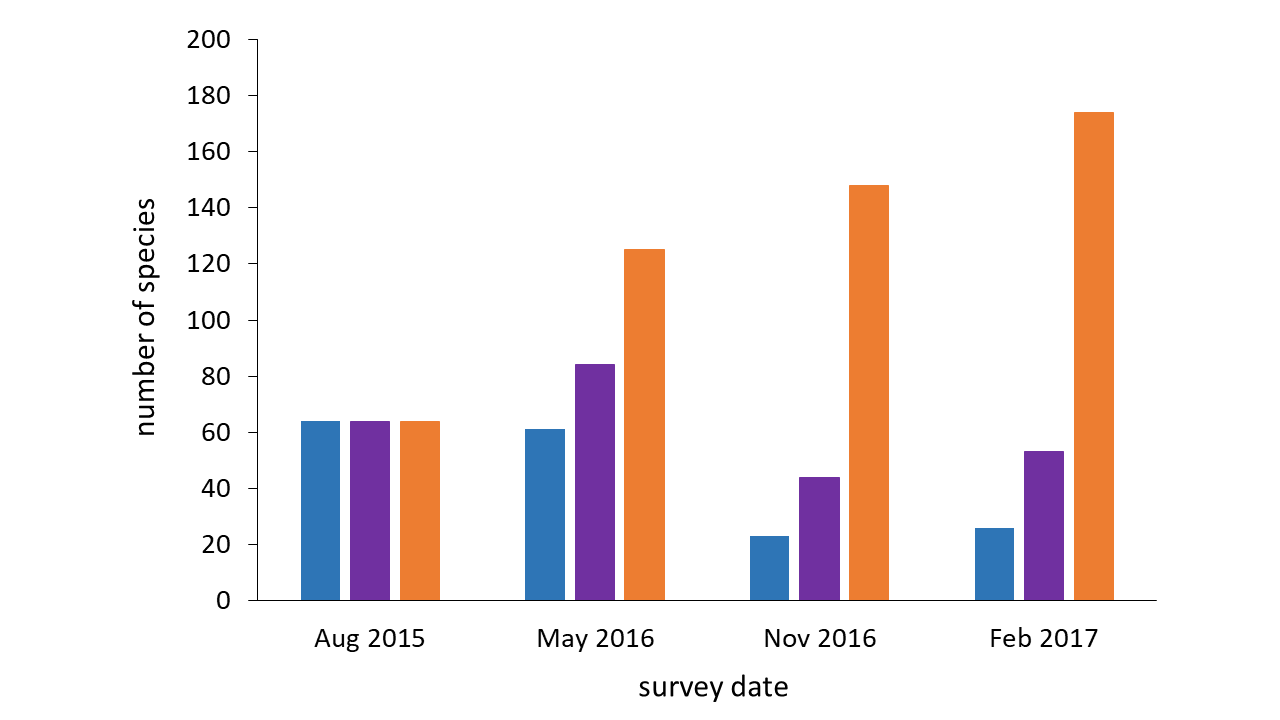
**
